# Cerebral small vessel disease as a possibly immune-related adverse event of immunotherapy in lung cancer patients: a retrospective study

**DOI:** 10.3389/fimmu.2025.1645549

**Published:** 2025-08-26

**Authors:** Na Wu, Dongmei Zhou, Xiaoyu Guo, Jia Liu, Jiafan Liu, Fan Liu, Xiaonan Wang

**Affiliations:** ^1^ Department of Gerontology and Geriatrics, The First Hospital of China Medical University, Shenyang, Liaoning, China; ^2^ Department of Medical Oncology, The First Hospital of China Medical University, Shenyang, Liaoning, China

**Keywords:** lung cancer, immune checkpoint inhibitors, cancer immunotherapy, neurological adverse event, cerebral small vessel disease

## Abstract

**Background:**

This clinical study aims to investigate the incidence of cerebral small vessel disease (CSVD) in lung cancer patients treated with ICIs and to analyze its risk factors by comparing the clinical features and laboratory tests in ICIs-treated lung cancer patients with or without CSVD.

**Methods:**

This retrospective study included 400 hospitalized patients from January 2018 to May 2024. All patients had confirmed lung cancer, received at least one cycle of ICIs, and underwent cranial MR imaging before and after ICIs treatment. Information from the medical records, including clinical features, MR imaging findings, laboratory tests, complications, treatment, and clinical outcomes, was extracted for analysis.

**Results:**

104 (26%) patients with CSVD were confirmed and 53.25% were aged≥65 years. Risk factors identified as independent predictors of CSVD included age (OR, 1.03), stage IV (OR, 2.87), and hyperlipidemia (OR, 1.02). In the CSVD group, FT_4_ levels decreased significantly between baseline and at the time of CSVD diagnosis, from 13.21 ± 4.56 pmol/L to 11.01 ± 2.11 pmol/L. TSH levels increased from 4.12 ± 0.46 pmol/L to 4.78 ± 1.13 pmol/L, cysteine C levels increased from 1.01 ± 0.98 mg/L to 1.29 ± 0.86 mg/L, PLR increased from 164.93 ± 27.86 to 171.27 ± 32.29 and SII rose from 774.28 ± 53.57 to 790.65 ± 68.34. All of them had no significance in the Non-CSVD group. Further Cox regression analysis showed that hypothyroidism (HR=2.38; 95% CI:1.89-5.04, P=0.005) was independent risk factors for CSVD. The incidence of hypothyroidism was 19.5% (78/400), and 43.6% (34/78) among them had CSVD. As predictors of CSVD, the cut point for FT_4_ was 11.84 pmol/L, and for TSH, it was 4.23 pmol/L. In Survival Analysis, CSVD did not show a significant impact on the median progression-free survival (PFS) and overall survival (OS) of lung cancer patients.

**Conclusion:**

This study found that CSVD may be a related adverse event of immunotherapy in lung cancer patients. In addition to age≥65 years, hyperlipidemia and stage IV, hypothyroidism, elevated cysteine C levels, and elevated systemic inflammatory markers such as PLR and SII were further associated with an increased risk of CSVD.

## 1 Introduction

Lung cancer is a malignant tumor with the highest incidence and mortality rates globally. Due to the 40% global tobacco consumption rate, and the high levels of ambient particulate matter pollution in developing countries, China is among the countries with a high incidence of lung cancer (1). Lung cancer has always been the leading cause of cancer mortality in China for both men and women (2). In recent years, immunotherapy, as an emerging strategy for cancer treatment, has gradually played a significant role in the treatment of lung cancer. Immune checkpoint inhibitors (ICIs) block immune checkpoints on the surface of tumor cells, such as Programmed Death Protein 1 (PD-1) and its ligand PD-L1, thereby enhancing the body’s anti-tumor immune response. Immunotherapy has achieved significant clinical outcomes in real-world studies, greatly improving patient survival rates (3, 4). Currently, there are 18 types of ICIs listed in China, 11 of which have been independently developed by China. Sintilimab, Tislelizumab, Toripalimab, and others have achieved good therapeutic effects in the treatment of lung cancer, thereby reducing the medical burden as part of their expenses can be covered by medical insurance (5–7). However, the widespread clinical application of ICIs is accompanied by a series of immune-related adverse events (irAEs). Neurologic irAEs include irMeningitis, irEncephalitis, irDemyelinating disease, irVasculitis, irNeuropathy, irNeuromuscular junction disorders and irMyopathy (8, 9). Previous studies suggest that the rate of progression of total aortic plaque volume was more than threefold higher with ICIs (10), and ICIs-related acute cerebrovascular events have been reported (11, 12).

The cerebral small vessels include arterioles, venules, and capillaries, which are important components of the cerebral vascular system. Cerebral small vessel disease (CSVD) is a class of diseases that affect the cerebral small vessels, which can manifest as white matter hyperintensities, lacunar infarcts, and other imaging features. Vascular endothelial dysfunction may be the underlying pathological alteration in CSVD (13). CSVD is explicitly age-related, once thought to be innocuous, but now recognized as the most important vascular contributor to dementia, and associated with clinical manifestations such as cognitive impairment and difficulty walking (14). Current research has identified that CSVD is related to a variety of risk factors, including hypertension, diabetes, and hyperlipidemia, among others (15). Cognitive dysfunction associated with ICIs has also attracted attention. Cancer-related cognitive decline is caused by multiple factors, including concomitant co-morbidities and various cancer treatments (16). A longitudinal study showed that among 240 non-small cell lung cancer patients treated with ICIs, significant deterioration was observed in TMT (psychomotor speed, executive function), HVLTi (verbal memory), and HVLTd (delayed recall) scores after 6 and 12 months of treatment (17). However, there is still a lack of systematic research on whether immunotherapy increases the risk of CSVD in lung cancer patients, which provides an important background for this study.

To evaluate the correlation between ICIs and CSVD, this study adopts a retrospective observational research method, analyzing large-scale clinical data to investigate the incidence of CSVD in lung cancer patients after receiving ICIs treatment, and conducts an in-depth analysis of its related risk factors.

## 2 Methods

### 2.1 Study design and data sources

**Figure 1:**
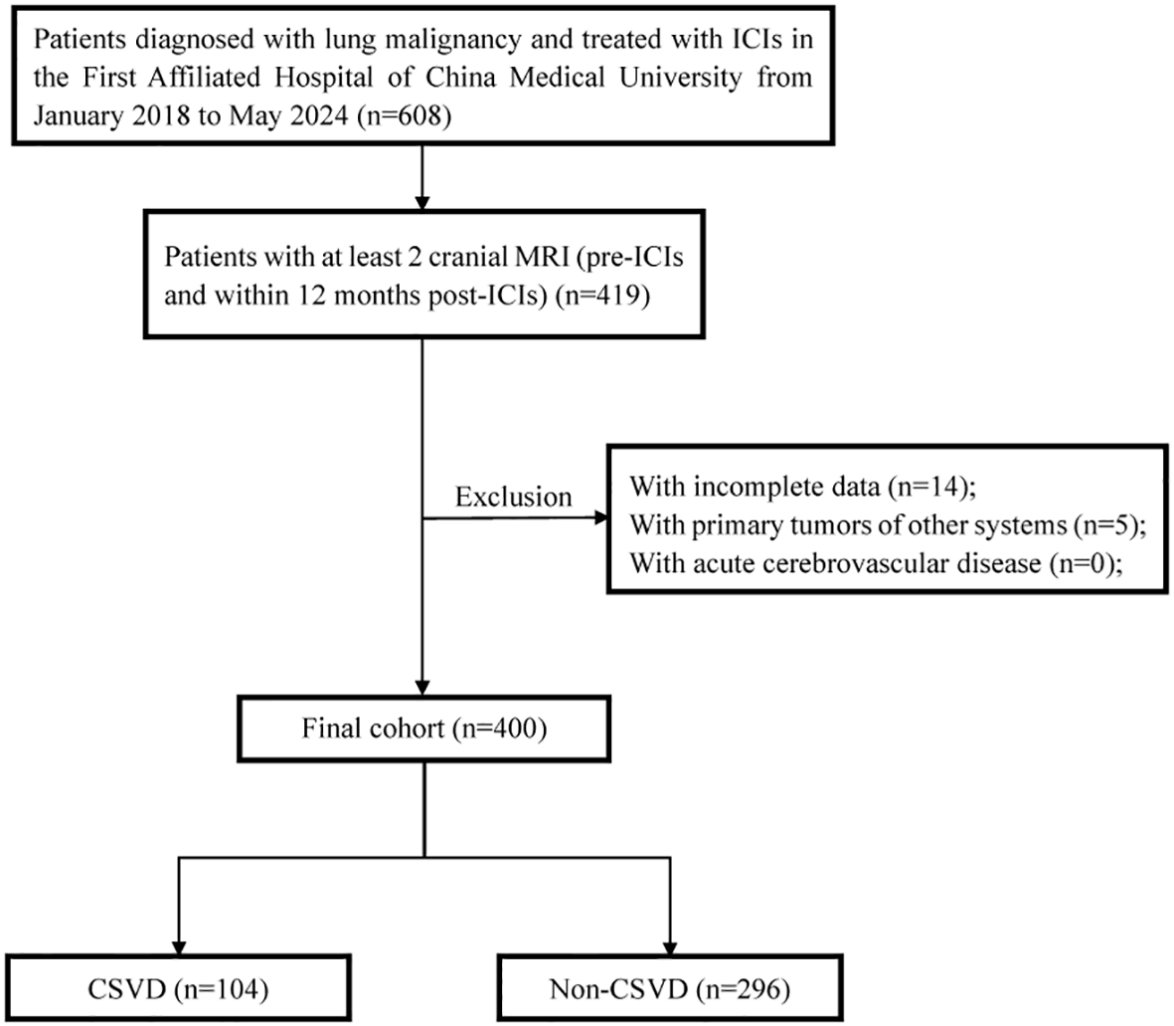
The flow chat of study design and patient inclusion. ICIs, immune checkpoint inhibitors; CSVD, cerebral small vessel disease.

608 lung cancer patients who received at least one cycle of ICIs therapy were collected between January 2018 and May 2024 at the First Hospital of China Medical University. Data was extracted from 12 months after the use of ICIs. All patients underwent cranial MRI imaging examination before receiving ICIs treatment, and we subsequently excluded cases meeting the following criteria: (a) age under 18 years, (b) a history of hematologic, (c) a history of primary brain cancer and cerebrovascular disease, (d) absence of brain MRI data (At least two cranial MRI images for analysis: pre-ICI and within 12 months post-ICI). 419 patients were included with cranial MR imaging before and after ICIs treatment; then, 19 patients were excluded for lack of response assessment. Ultimately, 400 patients were analyzed in this study. For further analysis, all patients were divided into two groups: those with CSVD and those without CSVD, based on cranial MR imaging. The flow chat of our study design is shown in **Figure 1**. Demographic, clinical, and survival data were retrieved from electronic medical records. All procedures performed in this study were in accordance with the Declaration of Helsinki (as revised in 2013). This study was approved by the Ethics Committee of the First Hospital of China Medical University (Project number: 2023-544-2).

### 2.2 Diagnosis and grouping of CSVD

In this study, CSVD was diagnosed using cranial MR imaging (3.0T) to obtain axial T1, T2-weighted, fluid-attenuated inversion recovery (FLAIR), and T2-weighted gradient echo (GRE) images. All MRI images were independently reviewed by two vascular neurologists; a third vascular neurologist adjudicated discordant findings. The imaging features of CSVD are white matter hyperintensity (WMH), lacunar infarction (LI), enlarged perivascular space (EPVS), cerebral atrophy (CA) and cerebral microbleed (CMB) (18). By comparing with pre-immunotherapy cranial MRI images, patients who had no baseline CSVD but developed new CSVD after treatment, as well as patients with baseline CSVD who exhibited new CSVD lesions or significant worsening of existing lesions post-treatment, were included in the CSVD group, while the remaining patients were assigned to the non-CSVD group.

### 2.3 Data collection

The demographic and clinical characteristics of lung cancer patients were collected from patient’s electronic medical records, including age (at initiation of ICIs), gender, body mass index (BMI), tumor histology type, initial cancer stage, immunotherapy regimens, line of ICIs therapy (e.g., first line, second line), cranial MR imaging history, and vascular risk factors (smoking, drinking, hypertension, diabetes, coronary heart disease and hyperlipidemia). Peripheral blood parameters included white blood cell count (WBC), neutrophilic count (NE), lymphocyte count (LY), hemoglobin count (HGB), platelet count (PLT), systemic immune-inflammation index ratio (SII: neutrophil count × platelet count)/lymphocyte count absolute monocyte count), neutrophil to lymphocyte ratio (NLR), platelet to lymphocyte Ratio (PLR), creatinine (Cr), cystatin C (Cys-c), estimated glomerular filtration rate (eGFR), uric acid (UA), C-reactive protein (CRP), lymphocyte subsets (CD4/CD8), free triiodothyronine (FT_3_), free thyroxine (FT_4_), thyroid stimulating hormone (TSH), cortisol (Cor) and adrenocorticotropic hormone (ACTH), fibrinogen (Fg) and D-dimer (D-D).

Due to subsequent requirements, the timeline of hypothyroidism in all patients and the progression of CSVD in those with hypothyroidism were also documented. Among patients with CSVD, we collected peripheral blood parameters at two time points: baseline (before ICI treatment) and at the time of CSVD diagnosis. In the non-CSVD group, these parameters were recorded at two time points: baseline data prior to the initiation of ICI therapy, and the final data within 12 months after ICIs treatment. The progression free survival (PFS) was calculated from the date of first administration of the ICIs until the progression of disease. The overall survival (OS) was calculated from the date of first administration of the ICIs until death or the last follow-up date (31 May 2025).

### 2.4 Statistical analysis

All analyses were performed using SPSS 26.0 (IBM, Armonk, NY, USA) and GraphPad Prism 9.0 (GraphPad Software, La Jolla, CA, USA). Two-side P values <0.05 were considered statistically significant. To describe general baseline characteristics, continuous variables data were expressed as mean ± standard deviation, and categorical variable data were summarized by frequency (%). The T-test, nonparametric test, or chi-square test were used to compare the baseline characteristics between groups, as appropriate. Logistic regression was performed to analyze the risk factors of CSVD. Selection of covariates in the multivariable models was based on univariate associations and biological relevance. An odds ratio (OR) with 95% confidence interval (CI) was reported for each covariate of interest. To address time-to-event outcomes, Cox proportional hazards regression was employed to identify CSVD risk factors. Univariable analyses of age, cancer stage, vascular risk factors and hypothyroidism yielded hazard ratio (HR) with 95% CI; significant predictors (*P* < 0.05) were retained in the final multivariable model with covariate adjustment. Proportional hazards assumptions were validated via Schoenfeld residuals (global *P* > 0.05), and multicollinearity was excluded (VIF < 2.0). The receiver operating characteristic (ROC) curve was performed to evaluate the diagnostic efficacy of data related to the occurrence of CSVD. The survival rates between the different groups were compared using the Kaplan-Meier method.

## 3 Results

### 3.1 MR image characters of CSVD

**Figure 2:**
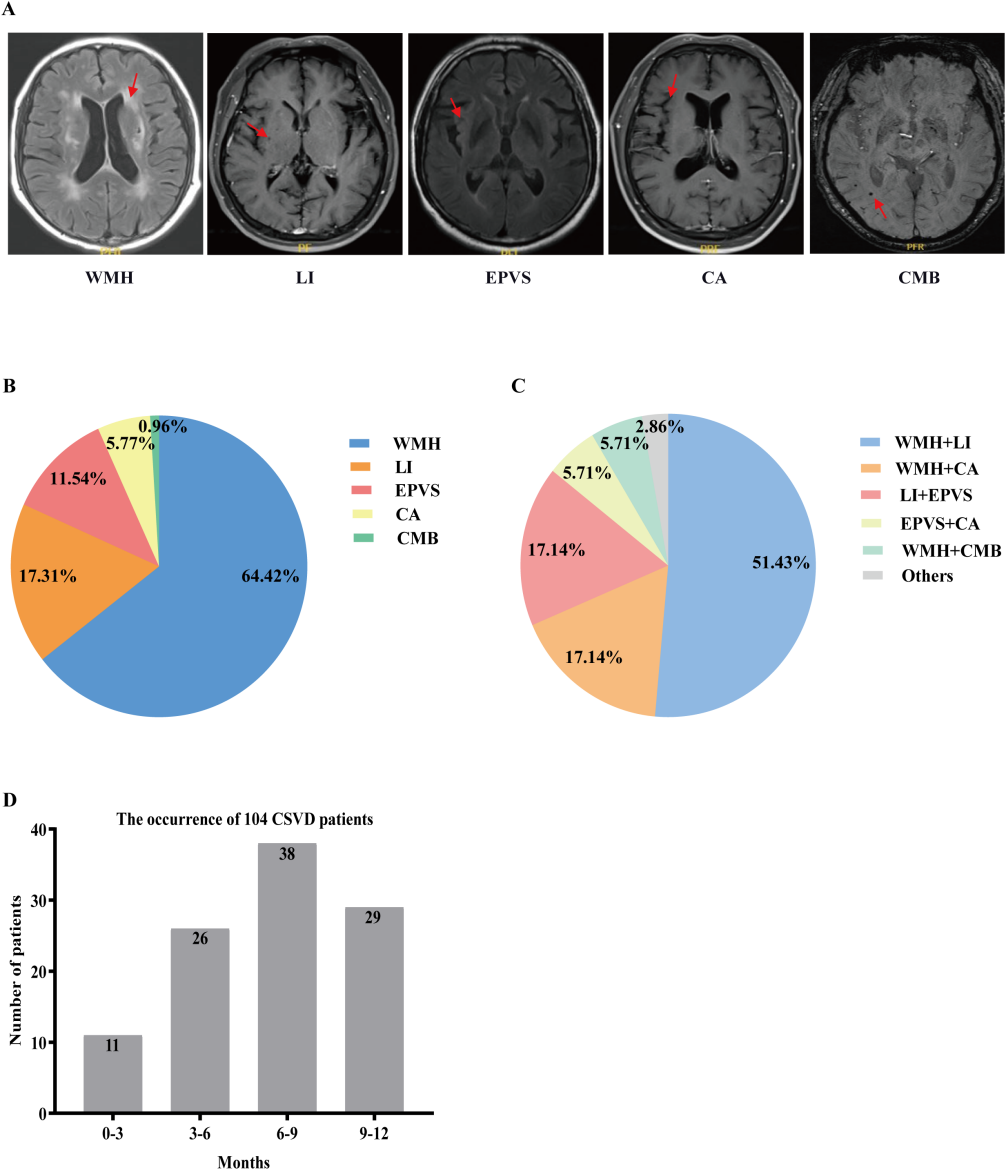
Imaging features of CSVD and distribution of (combinations of) CSVD manifestations. **(A)** Key imaging characteristics of CSVD. **(B)** Percentages of each lesion when one lesion is present. **(C)** Percentages of the combinations when two lesions are present. **(D)** The temporal distribution of CSVD following immunotherapy. CSVD, cerebral small vessel disease; WMH, white matter hyperintensity; LI, lacunar infarction; EPVS, enlarged perivascular space; CA, cerebral atrophy; CMB, cerebral microbleed.

The representative MR imaging examples were demonstrated in **Figure 2A**. **Figure 2B** showed the percentages of each lesion when one lesion was present: WMH (67/104, 64.42%), LI (18/104, 17.31%), EPVS (12/104, 11.54%), CA (6/104, 5.77%), and CMB (1/104, 0.96%). **Figure 2C** showed the percentages of the combinations when two lesions were present. We could see the most common combinations was WMH and LI (51.43%). Secondly, WMH and CA (17.14%), LI and EPVS (17.14%) with same proportion. Then, EPVS and CA (5.71%), WML and CMB (5.71%) also with same proportion. A total of 11 cases were diagnosed with CSVD within 0–3 months after ICIs treatment, peaking at 6–9 months with 38 cases. **Figure 2D** clearly demonstrates the temporal distribution of CSVD following immunotherapy.

### 3.2 Demographic and clinical characteristics

400 patients were included in this study and the incidence of CSVD in lung cancer patients treated with ICIs was 26% (104/400 patients). Slightly more than half were aged≥65 years, and 81.7% were male (**Table 1**). In China, the incidence of lung cancer caused by smoking is significantly higher in male patients than in females (19). The demographic and clinical characteristics of the enrolled patients were shown in **Table 1**. CSVD and Non-CSVD patients consisted of 26% (n = 104) and 74% (n = 296) of the entire lung cancer patients. 53.25% were aged≥65 years and 78.75% were male. There was no significant difference between groups with/without CSVD for age, gender, BMI, tumor histology, tumor stage, treatment data, vascular risk factors, baseline blood cell count, baseline eGFR and baseline TSH/FT_4_.

**Table 1:** Baseline characteristics of lung cancer patients treated with ICIs.

Variables	Total (N=400)	CSVD (n=104)	Non-CSVD (n=296)	P value
Age, years, n (%)				
<65	187 (46.75)	48 (46.15)	139 (46.96)	0.887
≥65	213 (53.25)	56 (53.85)	157 (53.04)	
Male, n (%)	315 (78.75)	85 (81.70)	230 (77.70)	0.389
BMI, kg/m^2^	23.01 ± 5.15	22.9 ± 4.87	23.13 ± 4.99	0.684
Type of cancer, n (%)				
Small-cell lung cancer	90 (22.50)	28 (26.90)	62 (20.94)	0.208
Non–small-cell lung cancer	310 (77.50)	76 (73.10)	234 (79.06)	
Cancer stage, n (%)				
≤ III stage	79 (19.75)	23 (22.12)	76 (25.68)	0.466
IV stage	321 (80.25)	81 (77.88)	220 (74.33)	
Immunotherapy, n (%)				
Monotherapy	73 (18.25)	18 (17.31)	55 (18.58)	0.758
Combination	327 (81.75)	86 (82.69)	241 (81.42)	
Line of ICIs therapy, n (%)				
First-line	179 (44.75)	45 (43.27)	134 (45.27)	0.723
≥ Second-line	221 (55.25)	59 (56.73)	162 (54.73)	
Brain radiation, n (%)	46 (11.50)	12 (11.54)	34 (11.49)	0.988
Vascular risk factors, n (%)				
Smoking	197 (49.25)	53 (50.96)	144 (48.65)	0.278
Drinking	56 (14.00)	14 (13.46)	42 (14.19)	0.313
Hypertension	86 (21.50)	23(22.12)	63 (21.28)	0.823
Diabetes	35 (8.75)	10 (9.62)	25 (8.45)	0.698
Coronary heart disease	18 (4.50)	6 (5.77)	12 (4.05)	0.348
Hyperlipidemia	126 (31.50)	34 (32.69)	92 (31.08)	0.631
Systolic blood pressure, mmHg )(kg/m^2^) (mmHg)	132.33 ± 12.56	131.29 ± 11.99	133.08 ± 11.03	0.140
Diastolic blood pressure, mmHg	81.81 ± 12.08	80.16 ± 12.55	82.43 ± 11.28	0.070
WBC, 10^9^/L	6.56 ± 2.30	6.44 ± 2.23	6.62 ± 2.35	0.556
NE, 10^9^/L	4.48 ± 2.11	4.39 ± 2.25	4.59 ± 2.13	0.503
LY, 10^9^/L	1.34 ± 0.74	1.29 ± 0.92	1.37 ± 0.68	0.511
PLT, 10^9^/L	241.84 ± 80.6	237.22 ± 92.19	248.35 ± 78.56	0.314
Cr, umol/L	64.58 ± 10.33	65.14 ± 10. 25	63.29 ± 10.49	0.188
Cys-c, mg/L	1.09 ± 0.89	1.16 ± 0.79	1.03 ± 0.97	0.295
eGFR, ml/min/1.73m^2^	91.28 ± 12.35	88.36 ± 13.78	90.51 ± 10.38	0.205
UA, umol/L	347.62 ± 98.79	353.68 ± 100.44	340.71 ± 105.56	0.343
CRP, mg/L	8.07 ± 5.21	8.57 ± 5.37	7.33 ± 6.49	0.140
CD4/CD8	1.76 ± 1.02	1.74 ± 1.55	1.78 ± 1.06	0.836
FT_4_, pmol/L	12.38 ± 4.56	11.79 ± 3.78	12.69 ± 2.11	0.054
FT_3_, pmol/L	4.56 ± 1.32	4.44 ± 1.09	4.62 ± 1.01	0.209
TSH, mIU/L	4.32 ± 0.76	4.40 ± 0.89	4.25 ± 0.67	0.182
ACTH, pg/ml	33.16 ± 11.79	32.54 ± 12.36	33.96 ± 11.26	0.385
COR, nmol/L	387.98 ± 121.43	385.98 ± 125.69	390.55 ± 116.79	0.785
Fg, g/L	3.24 ± 0.54	3.32 ± 0.60	3.21 ± 0.51	0.154
D-D, ug/ml	0.46 ± 0.37	0.47 ± 0.39	0.42 ± 0.31	0.339

ICIs, immune checkpoint inhibitors; CSVD, cerebral small vessel disease; BMI, body mass index; WBC, white blood cell; NE, neutrophile; LY, lymphocyte; PLT, platelet; Cr, creatinine; Cys-C, cystatin C; eGFR, estimated glomerular filtration rate; UA, uric acid; CRP, C-reaction protein; FT_4_, free thyroxine; FT_3_, free triiodothyronine; TSH, thyroid stimulating hormone; ACTH, adrenocorticotropic hormone; COR, cortisol; Fg, fibrinogen; D-D, D-dimer.

In the univariate logistic regression analysis, the results indicated that age (OR, 1.97; 95% CI: 1.16-2.79, P=0.025), stage IV (OR, 1.69; 95% CI: 1.24-2.01, P=0.040), and hyperlipidemia (OR, 2.37; 95% CI: 1.37-3.21, P=0.008) were associated with an increased risk of CSVD. Variables with a P-value ≤ 0.05 from the univariate logistic regression analysis were included in the multivariate logistic regression analysis. The results indicated that age (OR, 1.86; 95% CI: 1.22-2.81, P=0.036), cancer stage IV (OR, 1.81; 95% CI: 1.33-2.38, P=0.047), and hyperlipidemia (OR, 2.12; 95% CI: 1.34-3.16, P=0.013) were significantly and independently associated with the risk of CSVD (**Table 2**). Numerous studies have shown that age is a recognized independent risk factor for CSVD. Moreover, the severity and progression of CSVD increase with age (20, 21). Hyperlipidemia is a fatal risk factor for the development of stroke. Studies have shown that several common lipid abnormalities have a causal relationship with CSVD subtype infarction (22, 23). In this study, the results suggest that hyperlipidemia is an independent risk factor for CSVD in lung cancer patients undergoing immunotherapy.

**Table 2:** Risk factors for the development of CSVD in logistic regression analysis.

Variables	Univariable analysis		Multivariable analysis	
	*OR* (*95% CI*)	*P value*	*OR* (*95% CI*)	*P value*
Age, years, n (%)				
<65	1 (ref)	NA		
≥65	1.14 (1.05-1.23)	**0.026**	1.21 (1.13-1.37)	**0.036**
Male, n (%)	0.95 (0.64-1.33)	0.478		
BMI, kg/m^2^	0.99 (0.92-1.15)	0.681		
Type of cancer, n (%)				
Small cell lung cancer	1 (ref)	NA		
Non–small cell lung cancer	0.84 (0.72-1.05)	0.729		
Cancer stage, n (%)				
≤ III stage	1 (ref)	NA		
IV stage	1.69 (1.24-2.01)	**0.040**	1.81 (1.33-2.38)	**0.047**
Immunotherapy, n (%)				
Monotherapy	1 (ref)	NA		
Combination	1.05 (0.74-1.33)	0.857		
Line of ICIs therapy, n (%)				
First-line	1 (ref)	NA		
≥ Second-line	0.96 (0.64-1.41)	0.589		
Brain radiation, n (%)	1.24 (0.88-1.62)	0.203		
Vascular risk factors, n (%)				
Smoking	1.52 (0.91-2.18)	0.169		
Drinking	1.25 (0.76-1.71)	0.492		
Hypertension	1.65 (0.92-2.15)	0.361		
Diabetes	1.15 (0.79-1.37)	0.793		
Coronary heart disease	1.32 (0.96-1.71)	0.692		
Hyperlipidemia	2.37 (1.37-3.21)	**0.008**	2.12 (1.34-3.16)	**0.013**
Systolic blood pressure, mmHg	0.96 (0.88-1.17)	0.894		
Diastolic blood pressure, mmHg	0.99 (0.84-1.13)	0.813		
WBC, 10^9^/L	1.02 (0.88-1.14)	0.478		
NE, 10^9^/L	0.96 (0.58-1.31)	0.653		
LY, 10^9^/L	0.91 (0.98-1.25)	0.639		
PLT, 10^9^/L	0.81 (0.67-1.15)	0.893		
Cr, umol/L	0.98 (0.88-1.12)	0.791		
Cys-c, mg/L	1.05 (0.84-1.37)	0.764		
eGFR, ml/min/1.73m^2^	0.98 (0.90-1.18)	0.269		
UA, umol/L	1.02 (0.81-1.29)	0.961		
CRP, mg/L	1.11 (0.91-1.34)	0.279		
CD4/CD8	0.86 (0.68-1.14)	0.774		
FT_4_, pmol/L	0.93 (0.86-1.01)	0.132		
FT_3_, pmol/L	0.99 (0.77-1.24)	0.196		
TSH, mIU/L	1.03 (0.82-1.35)	0.347		
ACTH, pg/ml	0.97 (0.81-1.25)	0.429		
COR, nmol/L	0.99 (0.84-1.25)	0.791		
Fg, g/L	0.89 (0.76-1.05)	0.921		
D-D, ug/ml	0.95 (0.85-1.21)	0.812		

Bold values indicate P < 0.05 that is considered statistically significant; ICIs, immune checkpoint inhibitors; CSVD, cerebral small vessel disease; BMI, body mass index; WBC, white blood cell; NE, neutrophile; LY, lymphocyte; PLT, platelet; Cr, creatinine; Cys-c, cystatin C; eGFR, estimated glomerular filtration rate; UA, uric acid; CRP, C-reaction protein; FT_4_, free thyroxine; FT_3_, free triiodothyronine; TSH, thyroid stimulating hormone; ACTH, adrenocorticotropic hormone; COR, cortisol; Fg, fibrinogen; D-D, D-dimer. OR, odds ratios; CI, confidence interval.

### 3.3 Correlation of laboratory findings with CSVD

**Figure 3:**
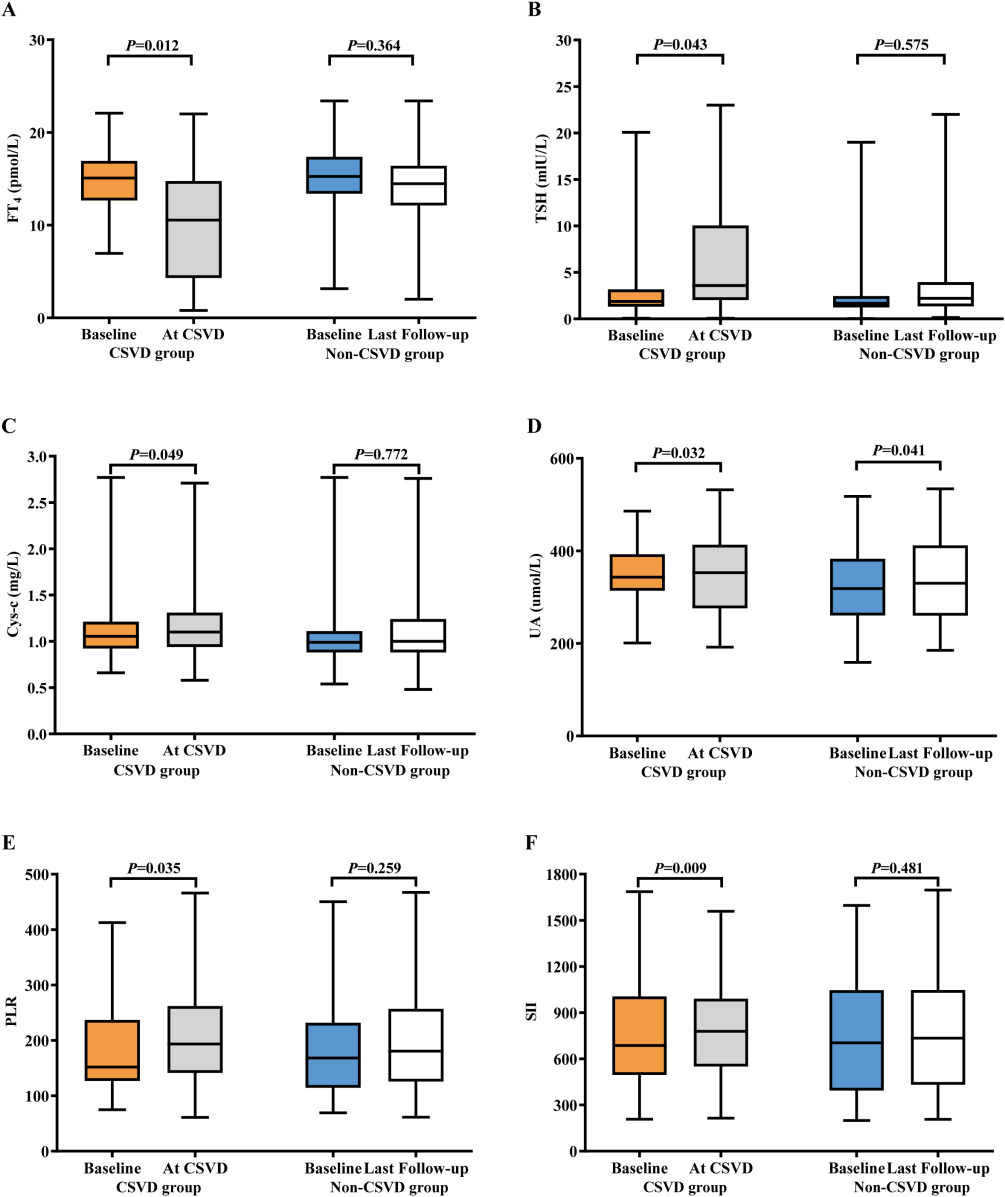
Bar plots of laboratory indicators in lung cancer patients with CSVD and non-CSVD at different times. **(A)** FT_4_. **(B)** TSH. **(C)** Cys-c. **(D)** UA. **(E)** PLR. **(F)** SII. CSVD, cerebral small vessel disease; UA, uric acid; Cys-c, cystatin C; FT_4_, free thyroxine; TSH, thyroid stimulating hormone; PLR, platelet to lymphocyte ratio; SII, systemic immune-inflammation index.

To clarify the specific changes in laboratory indicators during the occurrence of CSVD, we extracted laboratory data at the time of CSVD diagnosis and conducted a comparative analysis with baseline data. The results indicated no significant alterations in WBC, NE, LY, HGB, PLT, NLR, Cr levels, eGFR, CRP, CD4/CD8 ratio, FT_3_, Cor, ACTH, Fg, or D-D. However, there were notable changes in PLR, SII, Cys-c, UA, FT4, and TSH levels from baseline to the onset of CSVD, as shown in **Table 3**. A comparison of these six biomarkers was made between the CSVD group and the non-CSVD group. All six indicators showed no statistically significant differences when compared to baseline and final medication data (within 12 months after ICIs treatment) in the non-CSVD group (**Figure 3**). Apart from UA, the remaining five data points in the non-CSVD group did not show any statistically significant differences when comparing the baseline data with the final data (12 months after initiating immune checkpoint inhibitor therapy) (**Figure 3**).

**Table 3:** Comparison laboratory index in CSVD patients.

Variables	Baseline (n=104)	At CSVD (n=104)	P value
WBC, 10^9^/L	6.78 ± 2.13	6.52 ± 2.08	0.210
NE, 10^9^/L	4.56 ± 2.08	4.31 ± 1.88	0.193
LY, 10^9^/L	1.38 ± 0.55	1.29 ± 0.71	0.098
PLT, 10^9^/L	243.84 ± 71.6	236.38 ± 93.56	0.468
NLR	4.18 ± 3.26	4.63 ± 3.85	0.204
PLR	164.93 ± 27.86	171.27 ± 32.29	**0.035**
SII	774.28 ± 53.57	790.65 ± 68.34	**0.009**
Cr, umol/L	62.58 ± 10.12	65.34 ± 8.56	0.059
Cys-c, mg/L	1.01 ± 0.98	1.29 ± 0.86	**0.049**
eGFR, ml/min/1.73m^2^	91.31 ± 10.69	88.41 ± 12.14	0.066
UA, umol/L	323.68 ± 94.51	354.71 ± 102.56	**0.032**
CRP, mg/L	8.91 ± 6.21	9.33 ± 7.37	0.134
CD4/CD8	1.79 ± 1.02	1.72 ± 1.06	0.931
FT_4_, pmol/L	13.81 ± 2.56	11.05 ± 4.11	**0.012**
FT_3_, pmol/L	4.73 ± 1.45	4.51 ± 1.04	0.165
TSH, mIU/L	4.02 ± 0.46	5.78 ± 1.13	**0.043**
ACTH, pg/ml	32.12 ± 13.52	34.12 ± 15.22	0.689
COR, nmol/L	390.12 ± 120.21	386.72 ± 118.79	0.945
Fg, g/L	2.76 ± 1.01	2.66 ± 1.12	0.391
D-D, ug/ml	0.26 ± 0.55	0.31 ± 0.59	0.287

Bold values indicate P < 0.05 that is considered statistically significant; CSVD, cerebral small vessel disease; WBC, white blood cell; NE, neutrophile; LY, lymphocyte; PLT, platelet; NLR, neutrophil to lymphocyte ratio; PLR, platelet to lymphocyte ratio; SII, systemic immune-inflammation index; Cr, creatinine; Cys-c, cystatin C; eGFR, estimated glomerular filtration rate; UA, uric acid; CRP, C-reaction protein; FT_4_, free thyroxine; FT_3_, free triiodothyronine; TSH, thyroid stimulating hormone; ACTH, adrenocorticotropic hormone; COR, cortisol; Fg, fibrinogen; D-D, D-dimer.

Although no statistically significant differences were observed in inflammatory cell counts and non-specific biomarkers of inflammation (CRP and D-D), the composite biomarkers of systemic inflammation indices PLR and SII both exhibited an upward trend. Specifically, PLR increased from 164.93 ± 27.86 to 171.27 ± 32.29, with a P-value of 0.035; while SII rose from 774.28 ± 53.57 to 790.65 ± 68.34, with a P-value of 0.009. The final results indicated that FT_4_ levels decreased significantly from the baseline to when CSVD was diagnosed, from 13.21 ± 4.56 pmol/L to 11.01 ± 2.11 pmol/L, with a P-value of 0.012. A corresponding tendency in TSH levels was observed in the CSVD group, increasing from 4.12 ± 0.46 pmol/L to 4.78 ± 1.13 pmol/L, with a P-value of 0.043. Cys-c levels gradually increased from 1.01 ± 0.98 mg/L to 1.29 ± 0.86 mg/L (P = 0.049) in the CSVD group.

### 3.4 Potential risk factors for CSVD

To clarify the correlation between thyroid function and CSVD, hypothyroidism was further incorporated as a time-dependent covariate for Cox regression analyses. The results demonstrated that age ≥65 years (HR=1.31; 95% confidence interval CI: 1.05-1.81, P=0.043), stage IV cancer (HR=1.82; 95% CI: 1.42-2.96, P=0.015), hyperlipidemia (HR=1.65; 95% CI: 1.20-2.25, P=0.012), and hypothyroidism (HR, 2.38; 95% CI: 1.89-5.04, P=0.005) all exhibited significant independent correlations with CSVD (**Table 4**).

**Table 4:** Cox regression analysis of potential risk factors for CSVD.

Variables	Univariable analysis		Multivariable analysis	
	*HR* (*95% CI*)	*P value*	*HR* (*95% CI*)	*P value*
Age, years, n (%)				
<65	1 (ref)	NA		
≥65	1.43 (1.22-2.18)	**0.039**	1.36 (1.15-2.01)	**0.030**
Cancer stage, n (%)				
≤ III stage	1 (ref)	NA		
IV stage	1.85 (1.45-2.97)	**0.034**	1.69 (1.03-2.78)	**0.038**
Vascular risk factors, n (%)				
Smoking	1.62 (0.97-2.13)	0.117		
Drinking	1.20 (0.52-2.86)	0.612		
Hypertension	1.33 (0.50-3.50)	0.585		
Diabetes	1.14 (0.67-2.62)	0.390		
Coronary heart disease	1.15 (0.40-3.55)	0.836		
Hyperlipidemia	1.73 (1.21-2.34)	**0.011**	1.42 (1.02-1.95)	**0.034**
Hypothyroidism, n (%)	2.06 (1.57-4.22)	**0.026**	2.38 (1.89-5.04)	**0.005**

Bold values indicate P < 0.05 that is considered statistically significant; CSVD, cerebral small vessel disease; HR, hazard ratio; CI, confidence interval.

### 3.5 Association between ICIs-related hypothyroidism and CSVD

**Figure 4:**
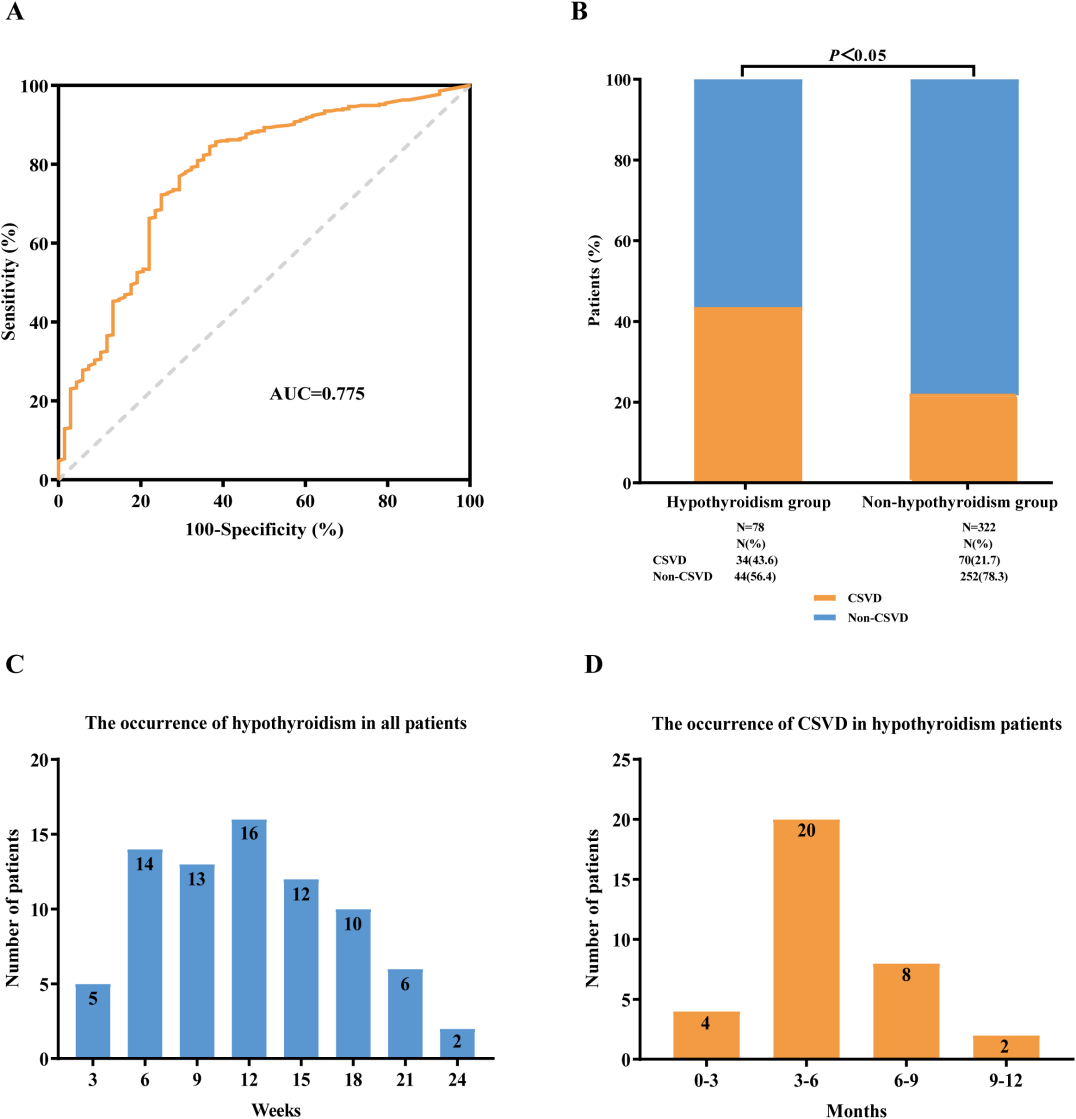
Association between immune checkpoint inhibitor-related hypothyroidism and CSVD. **(A)** ROC curve analysis of FT_4_. **(B)** The incidence of CSVD in lung cancer patients with hypothyroidism and non-hypothyroidism, respectively. **(C)** The occurrence of hypothyroidism in all lung cancer patients treated with ICIs therapy. **(D)** The occurrence of CSVD in ICIs-related hypothyroidism patients. ROC curve, receiver operating characteristic curve; AUC, area under the curve; FT_4_, free thyroxine; ICIs, immune checkpoint inhibitors; CSVD, cerebral small vessel disease.

Observations suggest that thyroid dysfunction may contribute to the occurrence of CSVD. Consequently, we compiled the timeline of hypothyroidism in all patients and analyzed the ROC curves to evaluate the predictive performance of FT_4_ and TSH levels as individual indicators. The optimal cutoff value for FT_4_ to distinguish the occurrence of CSVD was determined to be 11.84 pmol/L [AUC= 0.775 (95% CI 0.749-0.891), sensitivity = 70.4%, specificity = 80.5%, P = 0.028, **Figure 4A**]. The optimal cutoff value for TSH was determined to be 4.23 pmol/L [AUC = 0.548 (95% CI 0.672-0.805), sensitivity = 45.2%, specificity = 60.8%, P = 0.247] (**Supplementary Figure S1**).

During the period of ICIs treatment, we found 78 patients (19.5%) with confirmed hypothyroidism, and among these, 34 patients (43.6%) were confirmed to have CSVD. We observed that the incidence of CSVD was higher in the hypothyroidism group compared with the non-hypothyroidism group (43.6% versus 21.7%, P<0.05) (**Figure 4B**). The onset of hypothyroidism in all patients occurred from 3 to 24 weeks, with a peak at 12 weeks (**Figure 4C**). The incidence of CSVD in patients with hypothyroidism ranged from 0 to 12 months, peaking at 3 to 6 months (**Figure 4D**).

### 3.6 Association between CSVD and clinical outcomes

**Figure 5:**
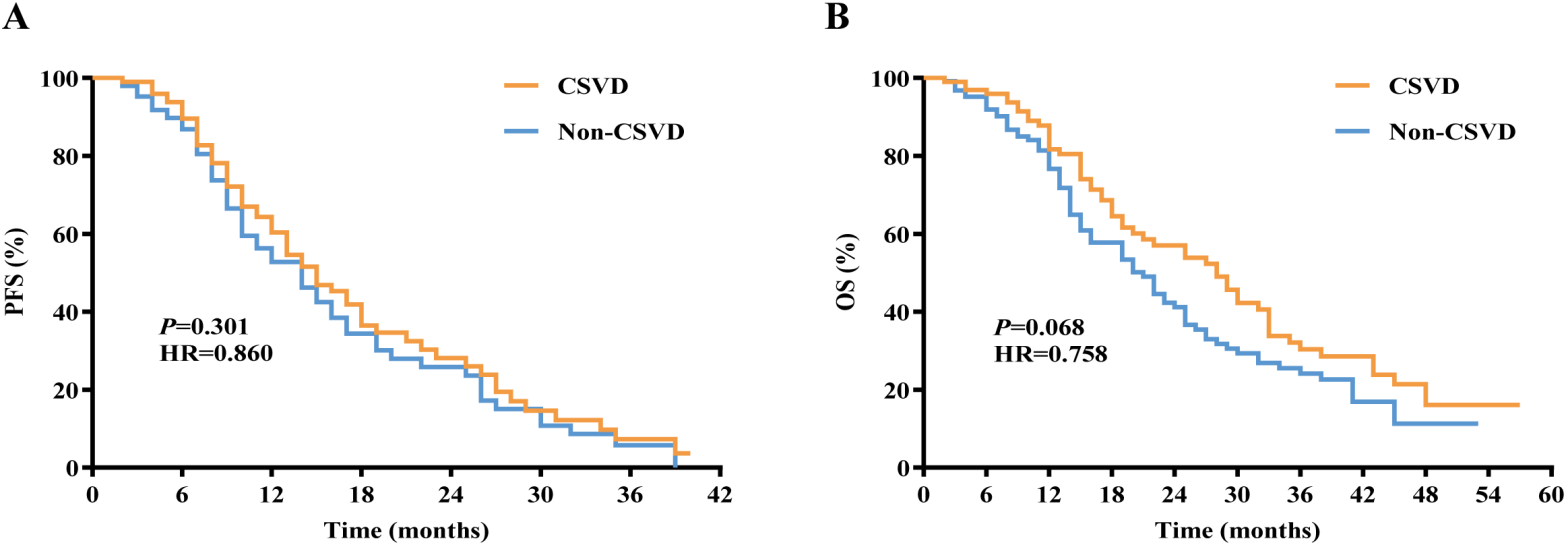
Kaplan-Meier curves for **(A)** PFS and **(B)** OS in lung cancer patients with or without CSVD. PFS, progression-free survival; OS, overall survival; CSVD, cerebral small vessel disease.

Among the 400 lung cancer patients, we compared the PFS and OS between the CSVD group and non-CSVD group. The Kaplan-Meier curve analysis revealed no significant difference in median PFS between the CSVD group and the non-CSVD group (14.52 months vs. 13.12 months, P = 0.882, **Figure 5A**), and similarly, no significant difference in median OS was observed (27.74 months vs. 23.69 months, P = 0.068, **Figure 5B**).

## 4 Discussion

Neurological adverse reactions to ICIs (n-irAEs) have garnered attention from neurologists and oncologists due to their severe consequences, prompting the need for early diagnosis and management (24). CSVD is a syndrome caused by various pathological changes in intracranial small blood vessels, commonly affecting the elderly. Its prevalence increasing with age (25), and often leading to cognitive dysfunction. The relationship between CSVD and ICIs is unclear. This retrospective study demonstrates, through the analysis of a large-scale sample, that lung cancer patients receiving immunotherapy exhibit a higher incidence of CSVD. Consistent with previous research findings, this study also confirms that age and hyperlipidemia are independent risk factors for CSVD. However, compared to the 20% incidence rate observed in elderly community populations, lung cancer patients undergoing immunotherapy exhibited a significantly higher incidence rate of 25% within a short-term period (12 months), despite having a younger average age (26). Poor staging may be related to cerebral small vessel damage caused by the late stage of the disease. Cystatin C is related to the severity and mortality of lung cancer, and some studies have proposed that it is also related to the prevalence of subclinical cerebral infarction (27–29). In this study, we similarly found that elevated cystatin C levels can increase the incidence of CSVD. Cystatin C is a cysteine protease inhibitor that has long been regarded as an ideal biomarker for evaluating renal function due to its nearly complete clearance by the kidneys. However, recent studies have shown that cystatin C plays a unique role in disease states such as atherosclerosis and cancer (30, 31). The specific mechanism by which cystatin C leads to CSVD remains unclear. Its potential mechanisms may involve vascular damage caused by the disruption of the balance between cystatin C and related cysteine proteases, as well as the participation of cystatin C as an inflammatory inducer in the inflammatory response process (32, 33).

Another novel finding in the present study is the level of FT_4_ and TSH is related to CSVD. Further data investigation and analysis revealed that a decline in thyroid function increases the incidence of CSVD. Thyroid dysfunction stands out as one of the most common endocrinopathies induced by ICIs therapy. Destructive thyroiditis is the pathophysiological basis shared by the most common patterns of thyrotoxicosis which was caused by T cell activation, alongside the involvement of various antibodies and cytokines (34–36). Thyroid hormones play a crucial role in the development of the brain and in maintaining brain function (37). Previous studies have suggested that thyroid dysfunction may accelerate the progression of CSVD (38). Hypothyroidism can lead to decreased cardiac output, which in turn causes insufficient microcirculatory perfusion in the brain, thereby affecting normal brain function (39). Other mechanisms include endothelial dysfunction and oxidative stress damage (40).The widespread application of ICIs in lung cancer patients may lead to an increased incidence of CSVD due to immune-related hypothyroidism. Although CSVD does not affect the PFS and OS of lung cancer patients, it may more significantly impact the patients’ quality of life. Maintaining stable thyroid function in patients is more conducive to treatment and reduces the incidence of CSVD. This study also provides the cut-off points for FT_4_ and TSH.

The findings of this study suggest that ICIs influence the function of cerebral small vessels through hypothyroidism. However, ICIs may also contribute to CSVD through more direct factors. In this study, it was discovered that the novel inflammatory markers SII and PLR, rather than NLR, were elevated in lung cancer patients with CSVD. The rise in these inflammatory indices indicates that immune dysfunction resulting from the use of ICIs may be linked to the development of CSVD. Various meta-analyses have suggested that elevated PLR and SII could be correlated with poorer PFS and OS among cancer patients undergoing ICIs treatment (41, 42). A large number of studies have shown that elevated SII levels are closely associated with severe CSVD burden and cognitive dysfunction (43), and higher SII levels are significantly correlated with WMH volume (44). SII reflects the systemic immune inflammatory state. In CSVD, systemic inflammation has a potential role in promoting the evolution and progression of WMH and microstructural damage (45). Endothelial dysfunction, microglial activation, atherosclerosis and blood-brain barrier injury are all key mechanisms of systemic inflammation-induced CSVD (46).

In summary, the research results indicate that when applying ICIs to treat lung cancer patients, it is necessary to pay special attention to the risk assessment of CSVD, which has important guiding significance for clinicians in formulating treatment plans and monitoring patient status. With the increasing dependence of lung cancer patients on immunotherapy, understanding the neurological complications that these patients may face after treatment can provide a basis for optimizing treatment plans, thereby improving the quality of life and survival rate of patients. This study identified risk factors for CSVD development after immunotherapy. Patients aged≥65 with pre-existing hyperlipidemia and stage IV demonstrated higher risks of CSVD when receiving immunotherapy. Hypothyroidism during immunotherapy was identified as an independent risk factor for CSVD. By monitoring and managing immune-related thyroid dysfunction, we can effectively reduce the incidence of CSVD, thereby improving patients’ quality of life.

This was a retrospective and single-center study with its own limitations. In this study, a high incidence of CSVD was observed among lung cancer patients undergoing treatment with ICIs, but we could not confirm that immunotherapy was an independent risk factor for CSVD. Additionally, female patients and large sample size were necessary for future study. This study only preliminarily explored the correlation between CSVD and immunotherapy for malignant tumors, and more data will be needed in the future to determine its deeper mechanisms and to advantage in clinic.

This study has determined the incidence of CSVD and its related risk factors in lung cancer patients receiving ICIs treatment. The study found that age, stage IV, hyperlipidemia and hypothyroidism were significantly and independently related to the risk of CSVD, while also revealing a correlation between PLR、SII and elevated cystatin C levels with CSVD. Although this study has certain limitations, it has revealed CSVD is a possibly related adverse event of immunotherapy, which has a certain guiding significance for future clinical diagnosis, treatment, and research.

## Data Availability

The original contributions presented in the study are included in the article/**Supplementary Material**. Further inquiries can be directed to the corresponding author.

## Supplementary material

Supplementary Figure 1 ROC curve analysis of TSH.
