# Supplementary figures and images for: Cerebral small vessel disease as a possibly immune-related adverse event of immunotherapy in lung cancer patients: a retrospective study
Source: Front Immunol. 2025 Aug 26;16:1645549. doi: 10.3389/fimmu.2025.1645549 (PMC12417442; doi:10.3389/fimmu.2025.1645549)

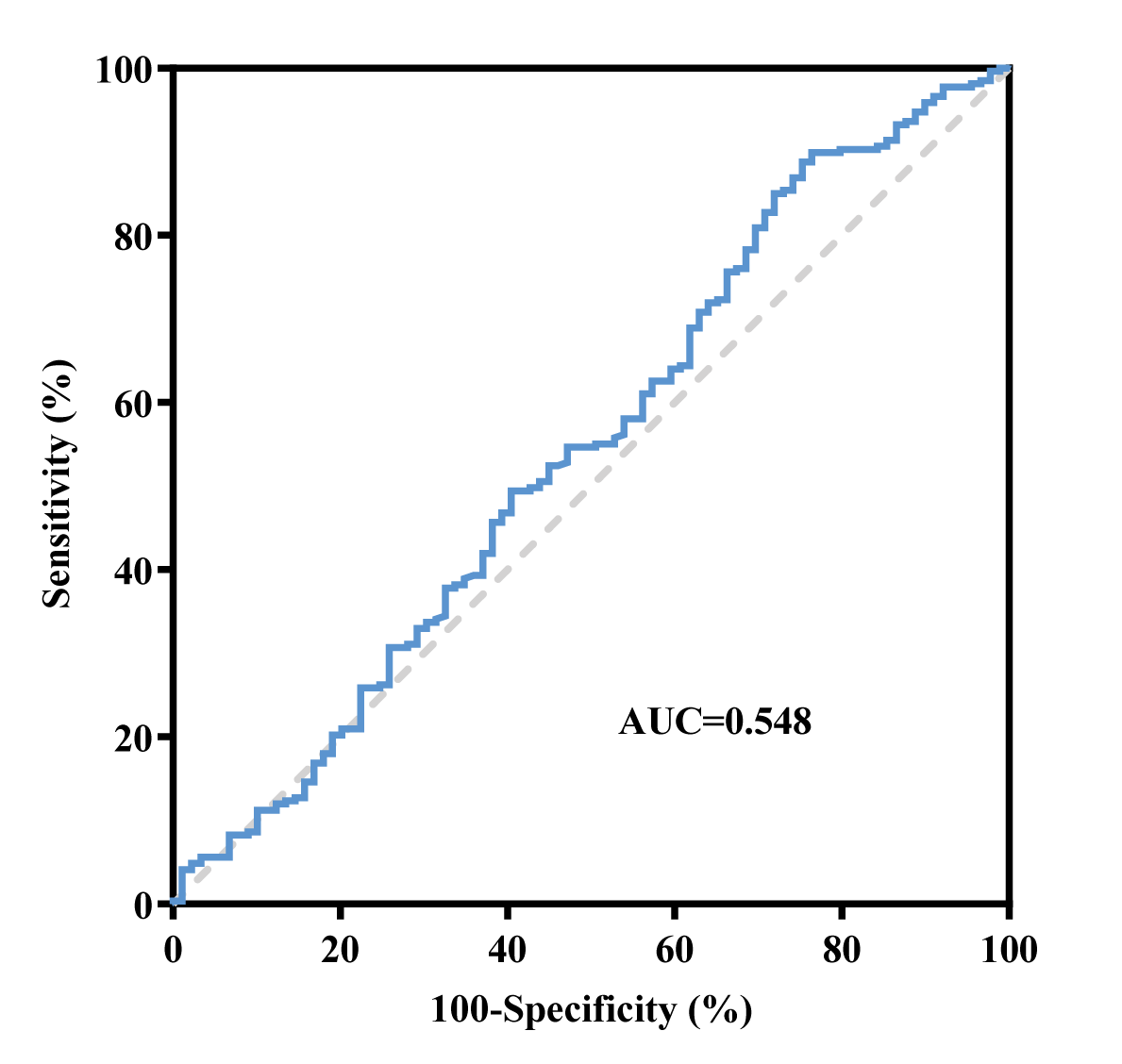

Supplement: Supplementary Figure 1 — ROC curve analysis of TSH. [file Image1.tif]
